# Essential Role of RAB27A in Determining Constitutive Human Skin Color

**DOI:** 10.1371/journal.pone.0041160

**Published:** 2012-07-23

**Authors:** Yasuko Yoshida-Amano, Akira Hachiya, Atsushi Ohuchi, Gary P. Kobinger, Takashi Kitahara, Yoshinori Takema, Mitsunori Fukuda

**Affiliations:** 1 Biological Science Laboratories, Kao Corporation, Haga, Tochigi, Japan; 2 Special Pathogens Program, National Microbiology Laboratory, Department of Medical Microbiology, Public Health Agency of Canada, University of Manitoba, Winnipeg, Manitoba, Canada; 3 Research and Development Global, Kao Corporation, Sumida-ku, Tokyo, Japan; 4 Laboratory of Membrane Trafficking Mechanisms, Department of Developmental Biology and Neurosciences, Graduate School of Life Sciences, Tohoku University, Sendai, Miyagi, Japan; University of Tennessee, United States of America

## Abstract

Human skin color is predominantly determined by melanin produced in melanosomes within melanocytes and subsequently distributed to keratinocytes. There are many studies that have proposed mechanisms underlying ethnic skin color variations, whereas the processes involved from melanin synthesis in melanocytes to the transfer of melanosomes to keratinocytes are common among humans. Apart from the activities in the melanogenic rate-limiting enzyme, tyrosinase, in melanocytes and the amounts and distribution patterns of melanosomes in keratinocytes, the abilities of the actin-associated factors in charge of melanosome transport within melanocytes also regulate pigmentation. Mutations in genes encoding melanosome transport-related molecules, such as MYO5A, RAB27A and SLAC-2A, have been reported to cause a human pigmentary disease known as Griscelli syndrome, which is associated with diluted skin and hair color. Thus we hypothesized that process might play a role in modulating skin color variations. To address that hypothesis, the correlations of expression of RAB27A and its specific effector, SLAC2-A, to melanogenic ability were evaluated in comparison with tyrosinase, using human melanocytes derived from 19 individuals of varying skin types. Following the finding of the highest correlation in RAB27A expression to the melanogenic ability, darkly-pigmented melanocytes with significantly higher RAB27A expression were found to transfer significantly more melanosomes to keratinocytes than lightly-pigmented melanocytes in co-culture and in human skin substitutes (HSSs) *in vivo*, resulting in darker skin color in concert with the difference observed in African-descent and Caucasian skins. Additionally, RAB27A knockdown by a lentivirus-derived shRNA in melanocytes concomitantly demonstrated a significantly reduced number of transferred melanosomes to keratinocytes in co-culture and a significantly diminished epidermal melanin content skin color intensity (ΔL* = 4.4) in the HSSs. These data reveal the intrinsically essential role of RAB27A in human ethnic skin color determination and provide new insights for the fundamental understanding of regulatory mechanisms underlying skin pigmentation.

## Introduction

Skin color is predominantly determined by the amount and types of melanin produced in melanosomes, melanocyte-specific organelles. In the process of mammal's skin color formation, melanin synthesis is launched by the melanogenic enzymes, tyrosinase, dopachrome tautomerase and tyrosinase-related protein-1, mutations in which cause hypopigmented or diluted color of skin [1], [2]. In addition to those enzymes, L-tyrosine and L-dihydroxyphenylalanine have also been shown to positively regulate melanin synthesis through the stimulation of tyrosinase activity [3]. Pigmented melanosomes are then transported towards the cell membrane at the dendrite tips of melanocytes via the intracellular transport system, anchored to the plasma membrane, and then transferred to neighboring keratinocytes [4]. After their transfer to keratinocytes, melanosomes are distributed around the nuclei by a microtubule-associated motor protein, dynein, to form supranuclear melanin caps which protect keratinocytes from the harmful effects of ultraviolet exposure [5], [6]. The substantial interaction between melanocytes and keratinocytes has been proposed by the anatomical finding that ∼36 viable keratinocytes surround each melanocyte to form a specialized cell group called the epidermal melanin unit [7], [8]. This ratio of the two cell types is consistently kept despite the extremely wide range of human skin color.

Melanogenesis is controlled by a wide range of molecular-based mechanisms in melanocytes and keratinocytes [8], which result in the variety of skin and hair pigmentation. Among them, melanocyte stimulating hormone (MSH) and its receptor, MC1R, which regulate melanogenic signaling via cAMPs, have been actively investigated. Genetic and cellular studies have revealed that MC1R polymorphisms contribute to the differences in the ultraviolet sensitivity and in hair and skin color intensity in several ethnic groups [9], [10]. Recently, melanocytes themselves have been reported to produce neurotransmitters, neuropeptides and hormones such as MSH, catecholamines, serotonin and melatonin, suggesting its plausible role in the regulation of sensory nerve endings in the epidermis [11].

There are many studies that have proposed mechanisms underlying ethnic skin color variations, whereas the processes from melanin synthesis in melanocytes to the transfer of melanosomes to keratinocytes are common among humans. It has been reported that tyrosinase activity in darker (African-descent) skin is significantly higher than in lighter (Caucasian) skin [12], [13] in spite of no difference in tyrosinase expression levels among them [14], [15]. Given that treatment with ionophore stimulators increase tyrosinase activity [16] and that a relatively lower melanosomal pH is observed in melanocytes from Caucasian skin compared to African-descent skin [17], melanosomal pH may be an important regulatory factor controlling enzymatic activity. Additionally, sodium/hydrogen exchangers (NHEs), which regulate intracellular pH and vesicular ATPase (V-ATPase), a vesicular proton pump, have been suggested to play roles in producing the variations of ethnic skin complexion [17], [18].

Differences in the amounts and distribution patterns of melanosomes in keratinocytes are also proposed to determine distinct ethnic skin colors. Architectural analysis showing numerous melanosomes throughout the epidermis in darker skin in contrast to only a few melanosomes in lighter epidermis has facilitated investigations on the differences in melanosome transfer [19]. It has been demonstrated that protease-activated receptor-2 (PAR-2), a seven transmembrane G-protein-coupled receptor, regulates phagocytosis in keratinocytes [20] and that darker skin exhibits a higher expression of PAR-2 compared to lighter skin [21]. Additionally, inhibition of PAR-2 has been reported to effectively lighten skin complexion [22]–[25], illustrating that PAR-2-mediated melanosome phagocytosis contributes to ethnic skin color differences. Apart from the numbers of melanosomes, keratinocytes from darker skin contain melanosomes that are predominantly distributed individually over the cytosol in contrast to lighter skin-derived keratinocytes which have their melanosomes in clusters [26], [27]. These different distribution patterns of melanosomes in the epidermis have been suggested to be regulated by keratinocytes not by melanocytes [28].

Coat color mutations in mice, such as *dilute* (*Myo5a^d^*), *ashen* (*Rab27a^ash^*) and *leaden* (*Mlph^ln^*), demonstrate impaired actin-associated melanosome transport which results in coat color dilution, indicating that melanosome transport within melanocytes is also necessary for the pigmentation of hair as well as skin [29]. Rab27a, the protein encoded at the *ashen* locus, is a small Ras-like GTPase belonging to the Rab family which contains about 60 members in charge of various types of membrane transport, such as vesicle fusion and docking of transport vesicles to specific target organelles and/or plasma membranes during secretory processes [30], [31]. It has been reported that Rab27a anchors melanosomes to the plasma membrane in collaboration with myosin Va, an actin-dependent motor protein encoded by the *dilute* locus, Slac2-a/melanophilin, a melanocyte-specific Rab27a effector encoded by the *leaden* locus and another effector Slp-2a in melanocytes [32]–[35].

On the other hand, mutations in the genes encoding MYO5A, RAB27A and MLPH are known to result in one of three subtypes of an autosomal recessive inherited human pigmentary diseases called Griscelli Syndrome (GS), which is mostly characterized as diluted pigmentation in skin and hair. A mutation in SLAC2-A exhibits a phenotypic change only in hypopigmentation [36], whereas mutations in the other two genes cause a wider range of phenotypes, such as neurological impairment (MYO5A) and immunodeficiency (RAB27A) [37]. Given that the physical interaction among these three factors to form a protein complex is required for melanosome transport [32]–[34], [38] and that the three subtypes of GS share a trait of hypopigmentation, it is reasonable to consider that the MYO5A-SLAC2-A-RAB27A complex plays a pivotal role in melanosome transport and in consequent cutaneous pigmentation in humans. However, it remains to be elucidated which melanosome transport-related factors substantially contribute to the determination of ethnic skin color differences.

In this study, we have evaluated the expressional correlation of several melanogenic- and/or melanosome transfer-related factors with the melanogenic activities in melanocytes from different ethnic backgrounds and subsequently validated their roles to elucidate the mechanisms underlying the ethnic skin color variations. The impact of RAB27A, which was selected as a possible candidate factor distinguishing ethnic skin color, on the amounts of melanocytic and epidermal melanin syntheses, on the transfer of melanosomes from melanocytes to keratinocytes and consequently on constitutive skin color was assessed using HSS technologies in collaboration with lentiviral-mediated shRNA technique to suppress RAB27A expression.

## Results

### Significantly higher numbers of melanosomes are transferred to keratinocytes in African-descent skin compared with Caucasian skin

**Figure 1:**
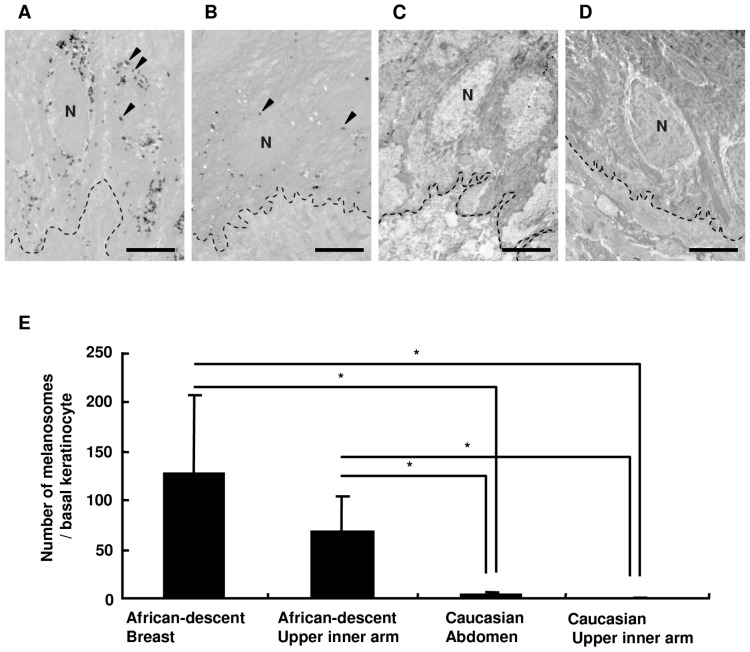
The number of melanosomes transferred to BKCs in the skin obtained from African-descent and from Caucasian individuals. Ultrastructural images of BKCs from African-descent subject-derived breast skin (A) and upper inner arm skin (B) and from Caucasian subject-derived abdominal skin (C) and upper inner arm skin (D). Fifty-one, thirty-one, forty-eight and thirty-one BKCs were imaged to calculate the numbers of melanosomes in those cells, respectively. N = nucleus, broken lines = basal membranes, and arrowheads point to melanosomes. Scale bars = 5 µm. The numbers of melanosomes transferred to BKCs is shown in (E). The values reported represent means ± SD. Statistical significance was evaluated by the Kruskal-Wallis test; asterisks indicate statistical significance (p<0.01).

The numbers of melanosomes transferred to basal keratinocytes (BKCs) between African-descent and Caucasian skins were compared to understand the ethnic differences in melanosome transfer. Breast and upper inner arm skins and abdominal and upper inner arm skins surgically prepared from two different African-descent subjects (Fig. 1A, 1B) and two different Caucasian patients (Fig. 1C, 1D), respectively, showed higher numbers of melanosomes within BKCs in the African-descent skin compared to the Caucasian skin, where transferred melanosomes in BKCs were hardly observed (Fig. 1C, 1D). The number of melanosomes per BKC in African-descent skin was found to be significantly higher compared to Caucasian skin (Fig. 1E).

### The expression of RAB27A mRNA and protein is significantly correlated with melanin content in melanocytes

**Figure 2:**
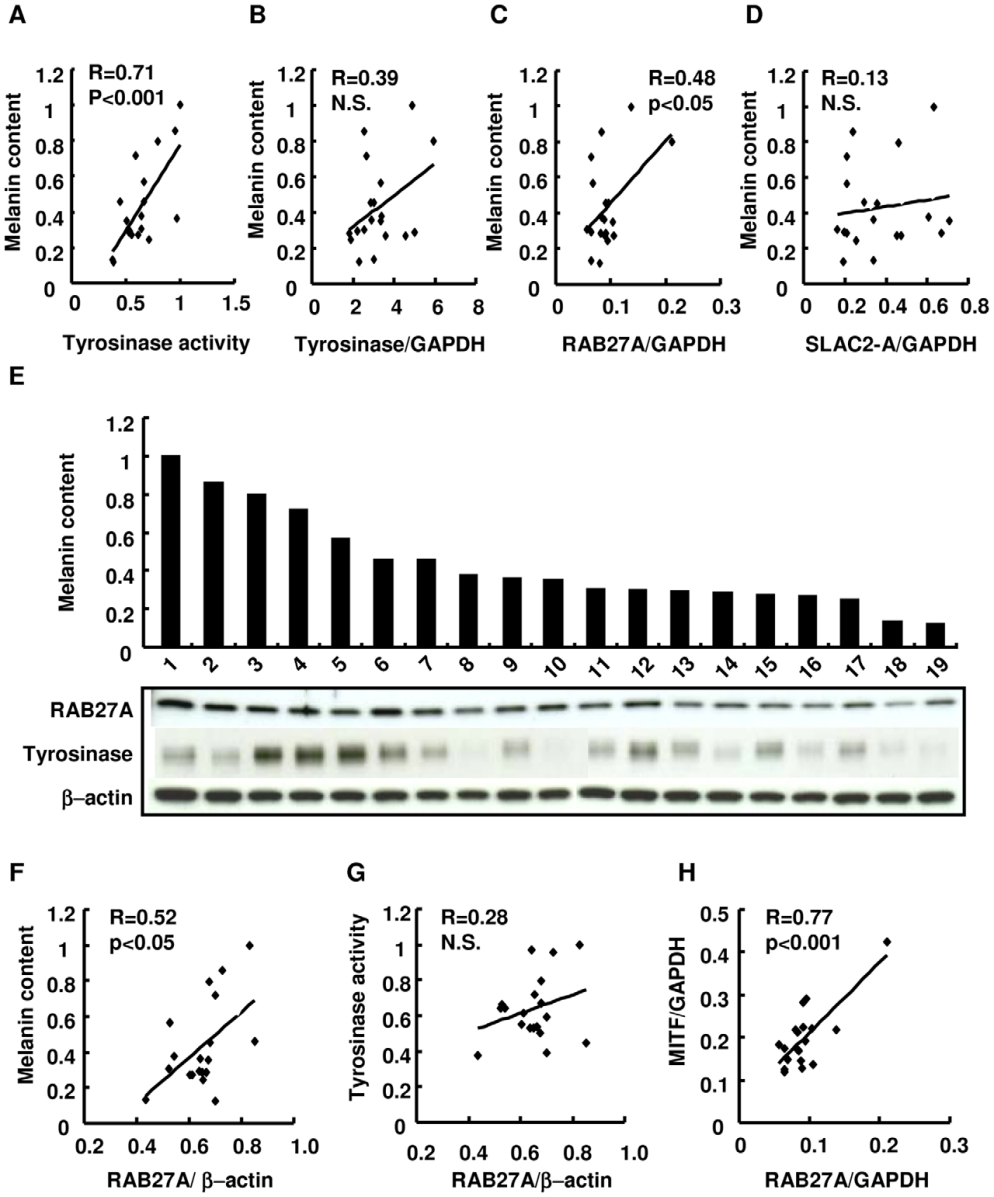
The RAB27A gene and protein expression in correlation with melanin content in cultured human melanocytes. Scatter plots demonstrate whether melanin contents correlate with tyrosinase enzymatic activity and/or with the levels of TYR, RAB27A and SLAC-2A transcript expression in cultured human melanocytes derived from 19 individuals (A–D). The expression levels of proteins of interest and melanin contents are presented using the aforementioned 19 cell lines of melanocytes (E). Melanin content in melanocytes is shown in a bar chart below which are the bands reflecting protein expression of RAB27A, tyrosinase and β-actin as marked. (D) Scatter plots demonstrate whether correlations are observed between the melanin content and/or the amount of RAB27A protein expression (F), between tyrosinase activity and the amount of RAB27A protein expression (H) and between the amount of transcript expression of MITF and RAB27A (H). The transcript expressions of TYR, RAB27A, SLAC2-A and MITF normalized by GAPDH expression are demonstrated (B–D, H). The amount of RAB27A protein expression was normalized by that of β-actin (F, G).

For the assessment of the contribution of well-known melanogenic- and/or intracellular melanosome transport-related factors in melanocytes to the ethnic skin color variations, the correlation of the enzymatic activity of tyrosinase and the gene expression of tyrosinase, RAB27A and SLAC2-A with melanin content were first examined using 19 commercially obtained cell lines of melanocytes with various melanin contents (Fig. 2A–D). Following the observation that tyrosinase enzymatic activity correlated with melanin content within melanocytes (Fig. 2A), the highest correlation between RAB27A mRNA expression level and melanin content in melanocytes was found (Fig. 2C). Consistently, Western blotting analysis also confirmed the correlation of RAB27A protein expression with melanin content using an identical set of cell lines (Fig. 2E, 2F). In addition, the expression level of RAB27A mRNA correlated with that of MITF mRNA in agreement with a previous finding (Fig. 2H) [39]. However, there was no correlation of melanin content with levels of tyrosinase protein expression (Fig. 2E) or RAB27A protein expression (Fig. 2G) in this study.

### More melanosomes from dark skin-derived melanocytes with higher RAB27A expression are transferred to keratinocytes than from light skin-derived melanocytes, resulting in higher melanin content in HSSs

**Figure 3:**
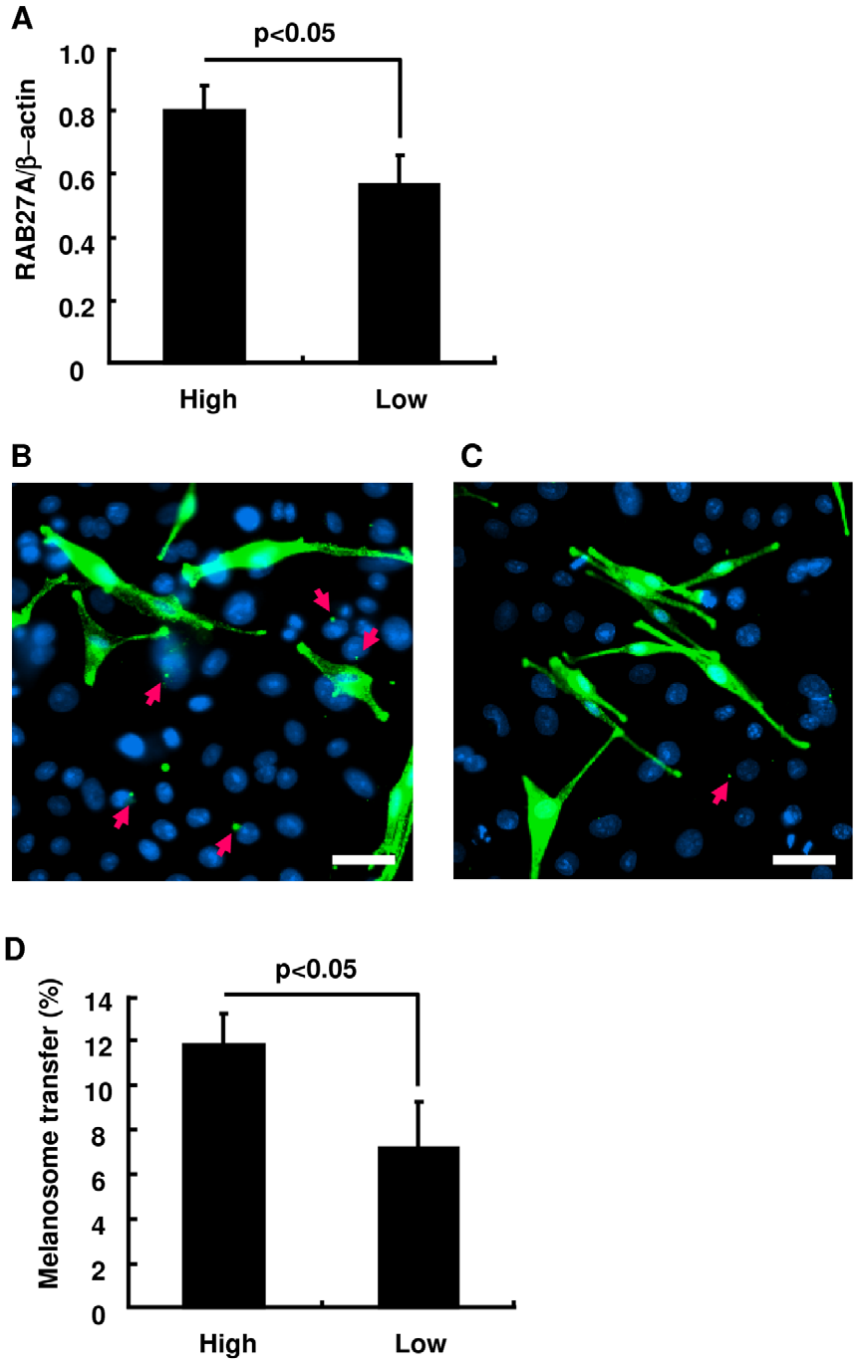
The ratio of keratinocytes with incorporated melanosomes is significantly higher when co-cultured with melanocytes expressing higher RAB27A amounts. (A) Melanocytes with higher RAB27A expression numbered 1, 4 and 6 and cells with its lower expression numbered 8, 16, 17 and 18 as explained in Figure 2 are divided into High and Low groups. Immunohistochemistry for melanosomal protein gp100 (green) in co-culture using High (B) or Low melanocytes (C) is demonstrated. Red arrows indicate melanosomes transferred into keratinocytes. Nuclei are stained blue with DAPI. Scale bars = 10 µm. (D) The ratio of keratinocytes harboring transferred melanosomes is compared when co-cultured with High and Low melanocytes. The values represent means ± SD. Statistical significance was evaluated by the Student's *t*-test, and a p value<0.05 is considered statistically significant.

**Figure 4:**
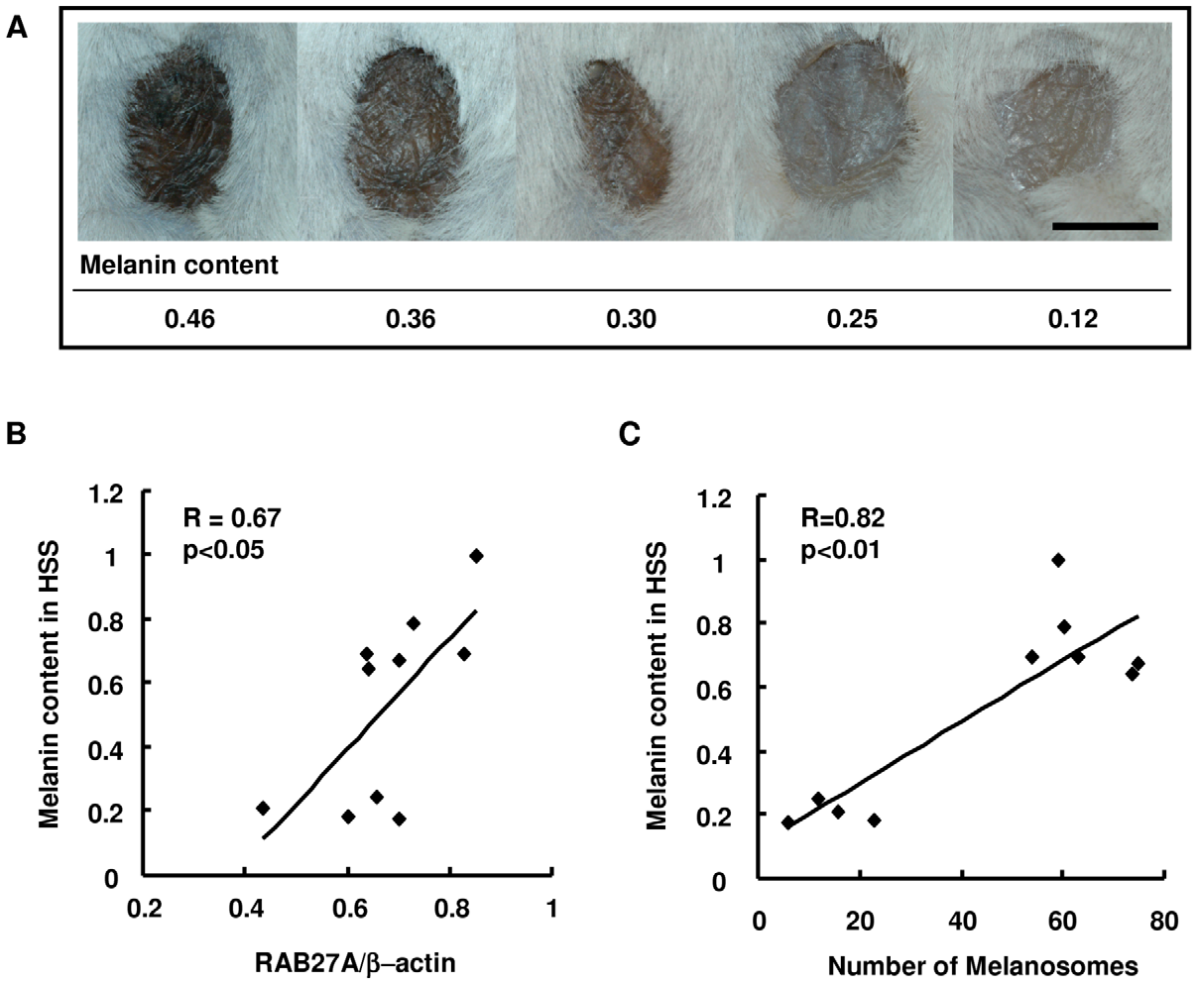
Melanin content in the epidermis of HSSs is correlated with the amount of RAB27A expression in the original cultured melanocytes. Surface images of HSSs composed of different lines of melanocytes with different expression levels of RAB27A together with same donor-derived keratinocytes and fibroblasts. Melanin contents in the original cultured melanocytes normalized by the amount of protein are shown at the bottom (A). Scale bar = 5 mm. Scatter plots indicate correlations between the expression level of RAB27A protein in the original cultured melanocytes and melanin contents in HSSs (B), and between the number of melanosomes in BKCs and melanin contents in HSSs (C).

To further examine the contribution of RAB27A expression to the amount of melanosome transfer and consequently to the intensity of skin pigmentation, melanocytes derived from three dark skin subjects and from four light skin subjects were co-cultured with keratinocytes from a light skin subject. The difference in RAB27A expression level between the two groups is shown in Fig. 3A. After the co-culture of keratinocytes with dark skin-derived melanocytes for 5 days, transferred melanosomes were easily detected with a specific antibody against the melanosome protein gp100 in adjacent areas of keratinocyte nuclei (Fig. 3B), in contrast to rarely observed melanosomes in keratinocytes co-cultured with light skin-derived melanocytes (Fig. 3C). The ratio of melanosome-incorporated keratinocytes to total keratinocytes was significantly higher when the keratinocytes were co-cultured with dark skin-derived melanocytes expressing higher levels of RAB27A (Fig. 3D). Following co-culture-based assessment, HSSs *in vivo* were generated by separately mixing 10 cell lines of melanocytes correlating their melanin contents with RAB27A expression level together with identical cell lines of light-skin derived keratinocytes and fibroblasts. The intensity of HSS surface color was found to be dominantly regulated by the ability to synthesize melanin in the original melanocyte populations before they were used in the HSS (Fig. 4A). Consistently, the melanin content in HSS epidermis correlated with the expression level of RAB27A in the melanocytes prior to the HSS generation (Fig. 4B) and with the number of melanosomes transferred to BKCs (Fig. 4C).

### Effect of suppressing RAB27A expression on melanosome transfer and on skin color

**Figure 5:**
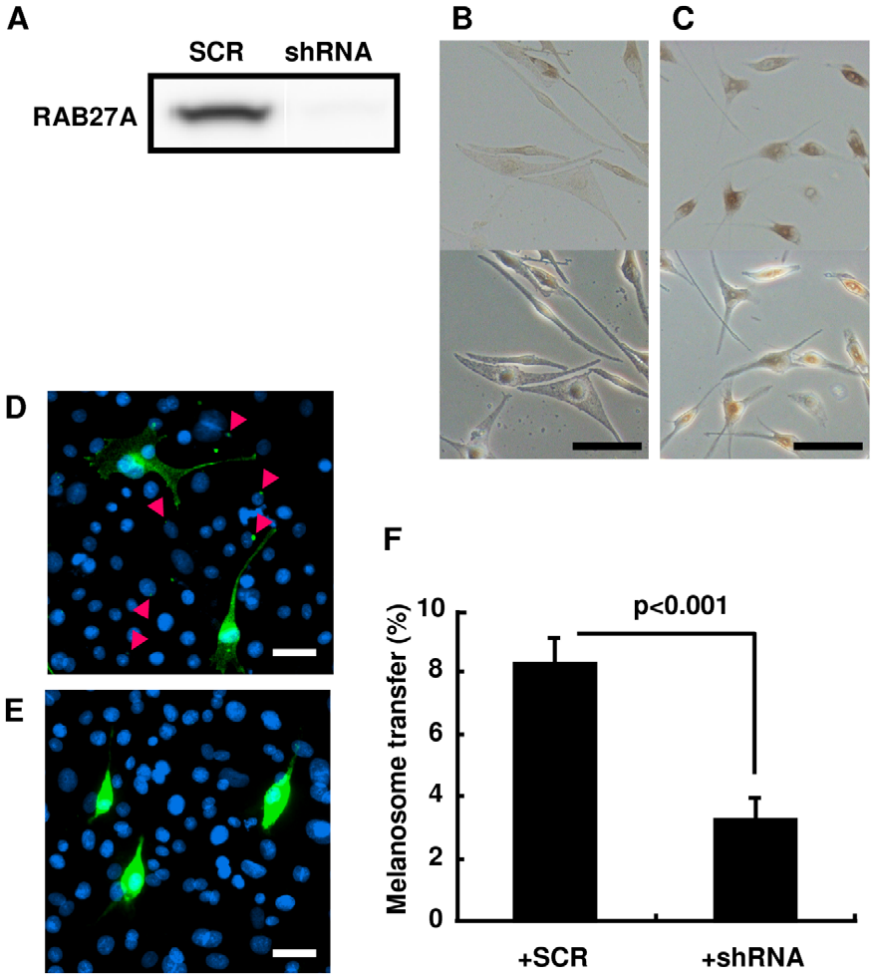
Knockdown of RAB27A expression in melanocytes by a lentivirus-driven shRNA significantly diminishes melanosome transfer into keratinocytes in co-culture. (A) Western blotting analysis of RAB27A in melanocytes treated with SCR or with RAB27A shRNA-expressing lentivirus (shRNA) following blasticidin-tolerant selection. Bright-field (upper panels) and differential interference contrast images (bottom panels) of melanocytes treated with SCR (B) or with shRNA (C). Scale bars = 10 µm. Immunohistochemistry for gp100 (green) in co-culture with melanocytes treated with SCR (D) or with shRNA (E) is demonstrated as described in Figure 3. Scale bars = 10 µm. (F) The ratios of keratinocytes harboring transferred melanosomes in co-cultures with melanocytes treated with SCR or with shRNA. The values represent means ± SD. Statistical significance was evaluated by the Student's *t*-test, and a p value of <0.05 is considered statistically significant.

**Figure 6:**
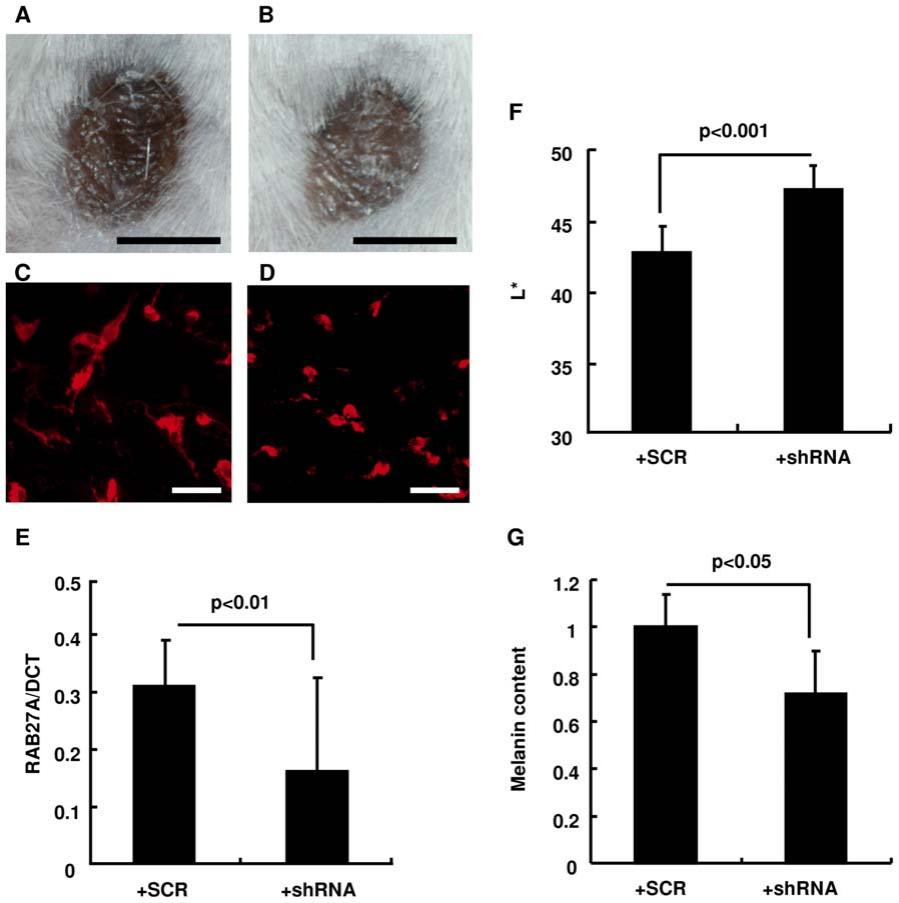
Reduced RAB27A expression in melanocytes significantly decreases the melanin content in the epidermis, resulting in a lighter skin surface color of HSS. HSSs were reconstructed by adding SCR- (A) or shRNA-treated melanocytes (B) to a mixture of normal keratinocytes and fibroblasts. Scale bars = 5 mm. Immunohistochemistry against melanosome specific protein TRP1 (red) in the HSS epidermis composed of SCR- (C) or shRNA-administered melanocytes (D) is demonstrated. Scale bars = 10 µm. The expression of RAB27A mRNA normalized to that of DOPAchrome tautomerase (DCT) (E), the surface color (F) and melanin content (G) are assessed in the HSS epidermis. Melanin content of the HSS epidermis composed of SCR-treated melanocytes was relatively expressed to be 1.0 (G). The values reported represent means ± SD. Statistical significance was evaluated by the Student's *t*-test and a p value<0.05 is considered statistically significant.

For the direct evaluation of the contribution of RAB27A expression to melanosome transfer to keratinocytes and to the resulting skin color, melanocytes treated with a lentivirus-driven RAB27A shRNA were used for melanocyte-keratinocyte co-cultures and the subsequent development of HSSs. Knockdown of RAB27A expression in melanocytes was confirmed by Western blotting analysis in cells treated with the RAB27A shRNA-lentivirus compared to cells treated with the scrambled RNA (SCR)-lentivirus after blasticidin selection for 3 wks (Fig. 5A). Even though there was no noticeable difference in cell shape between cells treated with either type of lentivirus (Fig. 5B, 5C), melanosome aggregation around the nuclei was observed only in melanocytes treated with the RAB27A shRNA-lentivirus (Fig. 5C) when compared to control cells treated with the SCR-lentivirus (Fig. 5B). Immunohistochemistry using an antibody specific for the melanosomal protein gp100 demonstrated that melanosome transfer to surrounding keratinocytes was remarkably reduced in co-cultures with RAB27A shRNA-lentivirus treated melanocytes (Fig. 5E). In contrast, substantial melanosome transfer was observed in keratinocytes co-cultured with SCR-lentivirus treated melanocytes (Fig. 5D). The ratio of melanosome-containing keratinocytes to total keratinocytes was significantly lower in keratinocytes co-cultured with melanocytes treated with the RAB27A shRNA-lentivirus compared to those co-cultured of the SCR-lentivirus treated cells (Fig. 5F). Following the evaluation with the co-culture system, HSSs *in vivo* were constructed using RAB27A-shRNA or SCR-lentivirus treated melanocytes for further investigation. Four weeks after the grafting of the cell mixture, the surface color of reconstructed HSSs composed of RAB27A shRNA-lentivirus treated melanocytes was lighter compared to those composed of melanocytes treated with the SCR-lentivirus (Fig. 6A, 6B). Immunostaining using an antibody specific for the melanosomal protein tyrosinase-related protein-1 and RT-PCR with a probe specific for RAB27A confirmed the melanosome aggregation within melanocytes and the reduced expression of RAB27A mRNA, respectively, in the epidermis when melanocytes treated with the lentiviral vector expressing shRNA for RAB27A were used (Fig. 6C–E). Consistently, HSSs composed of RAB27A shRNA-lentivirus transfected melanocytes had a lighter skin color (L* = 46.9) compared to those composed of melanocytes treated with the SCR-lentivirus (L* = 42.5) resulting in a significant difference in L* values (ΔL* = 4.4) (Fig. 6F). The data also showed that the melanin content in the epidermis was significantly lower in HSSs composed of melanocytes treated with the lentiviral vector expressing shRNA for RAB27A (Fig. 6G).

## Discussion

People whose ancestors originated from lower latitude areas tend to have darker skin color, whereas people whose ancestors were from higher latitude areas tend to have relatively lighter skin color, indicating that human skin color is one of the characteristics evolutionarily and geographically acquired to protect the skin from harmful ultraviolet radiation [40], [41]. Regarding such correlations, the Fitzpatrick Scale, which represents fair skin people to always or usually sunburn (Types I and II) and dark skin people to be only slightly or never sunburned (Types V and VI), was developed in 1975 to classify skin complexion and its tolerance to sunlight among different skin types and has been widely used for dermatologic research [42]. Apart from the paper demonstrating that the activity of melanosome transfer from melanocytes to keratinocytes is higher in darker skin than in lighter skin [19], we previously demonstrated that the origin of melanocytes predominantly determines the skin color rather than the keratinocytes in HSS systems *in vivo* [43]. Those findings extremely encouraged us to examine which melanocytic factors were predominantly engaged in the determination of skin color focusing on molecules involved in melanin transport processes within melanocytes where mature melanosomes initially bind to kinesin, a microtubule-associated motor protein, and are then directed to the cell periphery, followed by the detachment of melanosomes from microtubles and by their binding to actin filaments through the MYO5A-SLAC2-A-RAB27A complex [4]. In this study, we demonstrated a clear correlation between the expression level of RAB27A and melanin content and the significantly reduced melanosome transfer to keratinocytes and skin color intensity by the siRNA-driven down-regulation of RAB27A. These results consistently illustrated that RAB27A is in charge of the actin-associated melanosome transport within melanocytes and plays an essential role in determining ethnic skin color differences.

One important issue addressed in the current study refers to how RAB27A expression is regulated to impact the skin color intensity. It has been reported that the expression of RAB27A is regulated both directly by MITF and indirectly via cAMP [39], [44]. Consistent with those findings, the data presented here also suggest that MITF expression correlates with RAB27A expression. Additionally, MITF expression has been documented to be enhanced during UV-induced skin tanning accompanied with a remarkable increase in melanosome distribution throughout the epidermis in contrast to skin before UV exposure which contains melanosomes predominantly localized in BKCs [45]. Accompanied by the stimulated MITF expression, the enhancement of RAB27A expression would be harmonized with the increased melanin synthesis in melanocytes in order to effectively distribute melanosomes to keratinocytes and consequently to cover the epidermis for cutaneous photoprotection. In contrast, RAB27A mutations and gene knockdown have been reported not to affect melanin synthesis in melanocytes [46]. In *Xenopus laevis*, decreased expression of Rab27a and melanosome aggregation were observed when a dominant-negative Mitf was injected into their embryos, suggesting the involvement of Rab27a in melanosome transport in spite of the absence of subsequent melanosome transfer [47]. Since some species of amphibians, fish and reptiles can rapidly change their skin colors through mechanisms that translocate pigments and reorient reflective plates within chromatophores (pigment-containing and light-reflecting cells) which are largely responsible for modulating their skin color without changes in pigment production, RAB27A is a sort of vestigial machinery that is evolutionarily conserved to regulate skin color over the species.

It is also of interest to explore the various experimental systems to uncover the detailed molecular mechanisms underlying skin and hair homeostasis. We have previously established several efficient cutaneous and hair follicle gene transfer systems *in vivo* using vesicular stomatitis virus glycoprotein-pseudotyped lentiviral vectors [48]–[50]. However it remained a challenge to regulate the expression of specific genes only in targeted tissues and/or cells at will to examine long-term skin properties after grafting a cell mixture onto immunodeficient animals. In this study, we associated the *in vivo* HSS system with lentivirus-mediated gene knockdown for the first time and succeeded in developing HSSs containing melanocytes with continuously reduced expression levels of the gene of interest. This improved HSS technique could be a strong analytical tool to elucidate detailed molecular mechanisms of human skin disorders. Furthermore, the combination of epidermal and dermal cell preparations from newborn and from perinatal mice onto the backs of athymic nude mice has been applied to the identification of the cellular requirements for skin appendage formation [51]. The novel approach described here may also be useful for studies related to hair homeostasis.

Overall, our data show for the first time that RAB27A plays an important role in regulating the total amount of melanin in the epidermis and consequently in the determination of human ethnic skin color by controlling melanosome transport within melanocytes. The suppressed expression of RAB27A resulted in reduced melanin content in HSSs, indicating the impact of a molecule which is not involved in melanin synthesis but in vesicular transport in melanocytes, on the contribution to skin color formation *in vivo*. It has been reported that melanosome transfer is accelerated in regions of senile lentigos [52], [53]. Our findings provide new insights for the fundamental understanding of regulatory mechanisms that underlie skin pigmentation and provide a basis to develop an efficient strategy to treat cutaneous pigmentation disorders such as melasma, senile lentigo, seborrheic keratosis and vitiligo.

## Materials and Methods

### Materials

Normal human epidermal melanocytes (NHEMs), keratinocytes (NHEKs) and fibroblasts were obtained from Kurabo Co. (Osaka, Japan). Four to six week-old SCID mice were supplied by Oriental Bio-service Kanto Co. (Tsukuba, Japan). Abdominal and breast skins were received from a healthy 58-year-old Caucasian female undergoing abdominoplasty and from a healthy 40-year-old African-descent female undergoing breast reduction, respectively. Upper inner arm skin tissues were supplied from a healthy 32-year-old Caucasian female and a 36-year-old African-descent female by punch biopsy.

### Ethics statement

The collection of human skins was conducted according to Declaration of Helsinki principles and was approved by the Institutional Review Board of IntegReview (Austin, TX). Written informed consent was obtained from the subjects prior to the procedure. All mice were handled according to the guidelines of the Ethical Committee for Animal Experiments at the Kao Corp. (Tochigi, Japan). The protocol was approved by the Ethical Committee for Animal Experiments at the Kao Corp (Permit Number: N2009-0071A, N2009-0073A). All animal surgery was performed under isoflurane anesthesia, and all efforts were made to minimize suffering.

### Cell culture

NHEMs were maintained in Medium 254 (Kurabo) supplemented with 5 µg/ml insulin, 5 µg/ml transferrin, 3 ng/ml human recombinant basic fibroblast growth factor, 0.18 µg/ml hydrocortisone, 3 µg/ml heparin, 10 ng/ml phorbol 12-myristate 13-acetate (PMA), 0.2% (v/v) bovine pituitary extract (BPE), and 0.5% (v/v) fetal bovine serum (FBS) (Invitrogen) at 37°C in an atmosphere of 5% (v/v) CO_2_. NHEKs were maintained in Epilife medium (Kurabo) supplemented with 10 µg/ml insulin, 0.1 µg/ml human recombinant epidermal growth factor (EGF), 0.5 µg/ml hydrocortisone, 50 µg/ml gentamycin, 50 ng/ml amphotericin B, and 0.4% (v/v) BPE under the same conditions described above. Human skin fibroblasts were maintained in DMEM (Invitrogen) supplemented with 10% (v/v) FBS under the same conditions described above as well.

### Transmission electron microscopy (TEM)

For TEM analysis, human skins and HSS samples were fixed with 1/2 strength Karnovsky's fixation buffer, followed by 3 washes with 0.2 M sodium cacodylate buffer, and post-fixation with 1% osmium tetroxide containing 1.5% potassium ferrocyanide. After dehydration, tissues were embedded in Spurr's resin. Sections were obtained using a ULTRACUT S ultramicrotome (Leica Microsystems GmbH, Wetzlar, Germany) and were stained with uranyl acetate and lead citrate. Sections were observed and selected images were digitally registered using a H-7650 transmission electron microscope (Hitachi High-Tech, Tokyo, Japan).

### Grafting cells onto SCID mice

HSSs were prepared on SCID mice as previously described [39]. In short, melanocytes (1.0×10^6^), keratinocytes (6.0×10^6^), and fibroblasts (6.0×10^6^) were collected using 0.1% trypsin/EDTA, neutralized with an equal volume of FBS and then mixed and centrifuged for re-suspension. Silicone chambers (Renner, Darmstadt, Germany) were sutured onto the dorsal skins of mice and a suspension of combined cells was added into the 3 mm opening at the top of the chamber 3 hr after the installation of the chambers. One week after the implantation, the top part of each chamber was removed to increase air exposure and a wire net was attached to protect the HSSs from external damage. The remaining part of the chamber, including the base, was spontaneously released from the back of each mouse approximately four weeks after the implantation.

### Measurement of melanin content

Melanocytes were seeded at a concentration of 2.0×10^4^/cm^2^ in melanocyte culture medium without PMA and were cultured for 3 days. Cells were washed three times with PBS and were then dissolved with 2 M NaOH. The HSSs were surgically removed and incubated in 2 M NaBr to separate the epidermis from the dermis. The epidermis was soaked in 150 µl 2 M NaOH until completely dissolved. The absorbance of melanin at 405 nm was measured with a Model 550 Microplate Reader (Bio-Rad, Hercules, CA), and a melanin standard curve was prepared using synthetic melanin (Sigma-Aldrich, St Louis, MO). For the cultured melanocytes, melanin levels were quantitated and normalized by the amount of protein. The melanin level of cell line 1 was relatively presented to be 1.0. For the HSSs, melanin levels were quantitated per area of a punch biopsy 3 mm in diameter and the melanin level of the darkest HSS was relatively shown as 1.0.

### Tyrosinase activity measurement

Melanocytes were seeded in 12-well culture dishes at a density of 1×10^5^ cells/ml in melanocyte culture medium without PMA. After two days in culture, the cells were labeled overnight with 1.0 µCi/ml [3H]tyrosine and a 400 µl portion of the medium was applied to a water-equilibrated Sep-Pak AC-2 cartridge (Waters, Milford, MA). The released ^3^H_2_O was then eluted from the cartridge with 8 ml water and the eluate was transferred to a vial and mixed with scintillation fluid. The radioactivity was determined using a scintillation counter and was normalized by the amount of total protein, then the enzyme activity of cell line 1 (Fig. 2E) was presented to be 1.0.

### Melanocyte-keratinocyte co-culture assay

NHEKs were harvested at a concentration of 2.0×10^4^/cm^2^ with keratinocyte culture medium. After incubation for 24 hr, NHEMs were added at a concentration of 1.0×10^4^/cm^2^ in melanocyte culture medium without PMA and keratinocyte culture medium without human recombinant EGF and BPE at the ratio of 1∶2, followed by co-culture for another 3 days. Cells were washed and then fixed with ice-cold methanol and used for routine immunohistochemistry. The human gp100 antibody, HMB45 (DAKO, Produktionsvej, Denmark) at a 1∶40 dilution, was utilized for melanosome detection. Cell nuclei were stained with DAPI. Ten images were captured using a NIKON digital camera for each sample and the ratio of melanosome transfer was analyzed.

### Quantitative real time RT-PCR

Each HSS was pretreated with RNAlater (Qiagen, Valencia, CA) to stabilize total RNA and then the epidermal sheet was removed by incubation in 2 M NaBr. Total RNA from melanocyte cell cultures and epidermal sheets from HSSs was extracted using an RNeasy micro kit (Qiagen), followed by cDNA synthesis by reverse transcription of total RNA using a High Capacity cDNA Archive kit (Applied Biosystems, Foster City, CA). On-demand probes for human RAB27A, MITF and GAPDH in *Taq*Man Gene Expression Assays (Applied Biosystems) were used for real time quantitative RT-PCR performed in an ABI PRISM 7300 sequence detection system (Applied Biosystems).

### Western blotting analysis

Cultured melanocytes were washed with PBS and then solubilized in RIPA buffer (Sigma). A sample (5 µg) was separated using 12% SDS-polyacrylamide gel (Bio-Rad, Hercules, CA) after conventional extraction. A polyclonal RAB27A antibody from Santa Cruz (diluted at 1∶500), a monoclonal tyrosinase antibody from Upstate Biotechnology, Inc. (Lake Placid NY) (diluted at 1∶1000) and a monoclonal β-actin antibody from Sigma-Aldrich, Inc. (diluted at 1∶5000) were used as primary antibodies, followed by detection of the protein of interest using anti-mouse IgG horseradish peroxidase (HRP)-conjugated (GE Healthcare UK Ltd., Buckinghamshire, England) or anti-rabbit IgG HRP-conjugated (GE Healthcare UK Ltd.) as secondary antibodies.

### Vector design

Three RAB27A targeted BLOCK-iT™ shRNA vector sequences were designed and cloned into the pENTR™/U6 vector supplied from Invitrogen. The efficacy of shRNA was evaluated using the Target Screening System (Invitrogen) and then the sequence with the highest efficacy in gene silencing was selected and cloned into the pLenti6/BLOCK-iT-DEST™ destination vector (Invitrogen). pENTR™/U6-shRNA-Scramble Med GC (Invitrogen) was used for the negative control. Finally, the HIV vector encoding RAB27A shRNA was prepared by triple transfection of 293T cells with pCMVR8.2 packaging construct, pMD.G plasmid expressing VSV-G and pLenti6/BLOCK-iT-DEST™-RAB27A-shRNA or pLenti6/BLOCK-iT-DEST™ -shRNA-Scramble Med GC.

### Administration of the lentiviral vector encoding RAB27A shRNA into cultured melanocytes

NHEMs were seeded at a concentration of 2.0×10^4^/cm^2^ one day before the transfection. The melanocyte culture medium was replaced with fresh medium without PMA, followed by transfection with the VSV-G-pseudotyped lentiviral vector encoding RAB27A shRNA or the Scrambled-RNA at a concentration of 3.0×10^5^ TUml^−1^ in the presence of 6 µg/ml polybrene. On the following day, the transfection medium was replaced with fresh culture medium without PMA and cells were cultured for another 24 hr. Blasticidin (10 µg/ml) was then added and the medium with blasticidin was replaced every three days for three to four weeks until the cells were used for further analysis.

### Statistics

The level of significance of differences was analyzed by Kruskal-Wallis test for the number of melanosomes within basal keratinocytes between ethnic groups and by Student's t-test for other comparative analyses. The relationships between two variables were determined by Pearson's correlation coefficient and the significance of the slope of the regression line is determined from the t-statistic. A p value<0.05 is considered statistically significant.
